# Parasitic thyroid nodules in patient with nontoxic multinodular goiter: a case report

**DOI:** 10.1186/1752-1947-8-66

**Published:** 2014-02-20

**Authors:** José Rildo Fernandes de Oliveira Filho, Tales Rubens de Nadai

**Affiliations:** 1 Americo Brasiliense State Hospital, Alameda Aldo Lupo, 1260, 14820-000, Américo Brasiliense, SP, Brazil; 2 Department of Surgery and Anatomy, Ribeirão Preto School of Medicine, University of São Paulo, Avenida Bandeirantes, 3900, 14048-900 Ribeirão Preto, SP, Brazil

**Keywords:** Thyroid, Parasitic nodule and multinodular goiter

## Abstract

**Introduction:**

The presence of benign thyroid tissue that is located on the side of the neck is extremely rare and not related to the development of the thyroid, and it is difficult to differentiate it from thyroid carcinoma metastasis.

The parasitic thyroid nodule occurs when thyroid tissue located in the lateral neck has no relationship or association with the lymph nodes, and may be defined as a thyroid nodule entirely separate from the thyroid or attached to it by a narrow pedicle, presenting the same histology and in the same facial plane as the thyroid, and should not be associated with lymph nodes.

**Case presentation:**

A 40-year-old Brazilian man without significant past medical history presented with a large volume multinodular thyroid goiter that caused deformity and symptoms suggestive of cervical spine compression. He underwent a total thyroidectomy. His thyroid function was normal. Ultrasonography showed a heterogeneous thyroid nodule measuring 3.7cm to the right from midline and 3.3cm to the left from midline that was associated with two nodules in the left submandibular area measuring 1.43cm and 1.52cm.

Fine needle aspiration confirmed the benign nature of the gland and thyroid tissue etiology of the two submandibular nodules, located in level II of the neck. Since the ectopic thyroid tissue in his lateral neck was suggestive of metastasis of occult primary thyroid carcinoma, the patient underwent a total thyroidectomy plus a left modified radical neck dissection with preservation of level I. The diagnosis of multinodular goiter associated with two parasitic thyroid nodules was confirmed by immunohistochemistry.

**Conclusions:**

We conclude that the parasitic thyroid nodule should be included in the differential diagnosis of lateral neck masses. The diagnosis and differentiation of these nodules from metastatic adenopathies of differentiated thyroid carcinoma has important therapeutic and prognostic implications, and can lead to avoidance of unnecessary surgeries.

## Introduction

Ectopic thyroid tissue is a failure of migration of the thyroid during the embryonic period [1]. It can be present anywhere, from the foramen cecum, which is at the base of the tongue, to the normal position of the thyroid, which is between the second and fourth tracheal rings [1]. Lesions are usually midline, and this position is the most frequent presentation of ectopic thyroid tissue, presenting in 90% of cases [2]. The terms, accessory thyroid gland or tissue, have been used in these instances.

The presence of benign thyroid tissue located on the side of the neck is extremely rare and not related to the development of the thyroid, and it is difficult to differentiate from thyroid carcinoma metastasis [3].

The parasitic thyroid nodule occurs when thyroid tissue located in the lateral neck has no relationship or association with the lymph nodes, and may be defined as a thyroid nodule entirely separate from the thyroid or attached to it by a narrow pedicle, presenting the same histology and in the same facial plane as the thyroid, and should not be associated with lymph nodes [1]. We report the case of a parasitic thyroid nodule in a patient with multinodular goiter that simulated metastasis of an occult primary thyroid carcinoma.

## Case presentation

**Figure 1:**
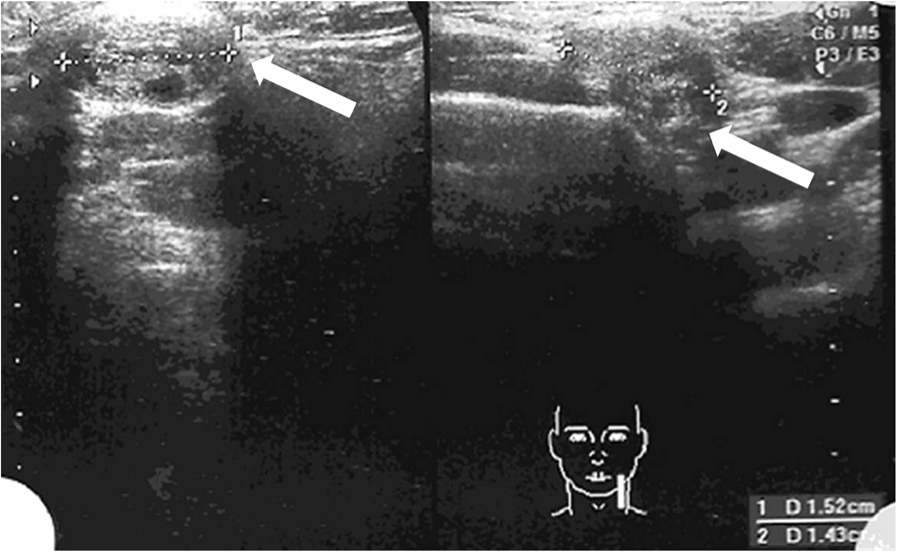
**Neck ultrasonography.** The arrows show two submandibular nodes with thyroid tissue.

A 40-year-old Brazilian man without significant past medical history presented to our institution in 2011 with a large volume multinodular thyroid goiter that caused deformity and symptoms suggestive of cervical spine compression. He underwent a total thyroidectomy. His thyroid function was normal. Ultrasonography showed a heterogeneous thyroid nodule measuring 3.7cm to the right from midline and 3.3cm to the left from midline that was associated with two nodules in the left submandibular area measuring 1.43cm and 1.52cm (Figure 1).

Fine needle aspiration confirmed the benign nature of the gland and the thyroid tissue etiology of two submandibular nodules located in level II in his neck. The thyroglobulin levels were not measured in fine needle aspiration. A frozen section of two submandibular masses was performed before thyroidectomy. As a result, ectopic thyroid tissue was noted, but the pathologist did not exclude the possibility of metastasis of occult primary thyroid carcinoma. Our patient underwent a total thyroidectomy, plus a left modified radical neck dissection with preservation of level I. The diagnosis of multinodular goiter associated with two parasitic thyroid nodules was confirmed by immunohistochemistry.

**Figure 2:**
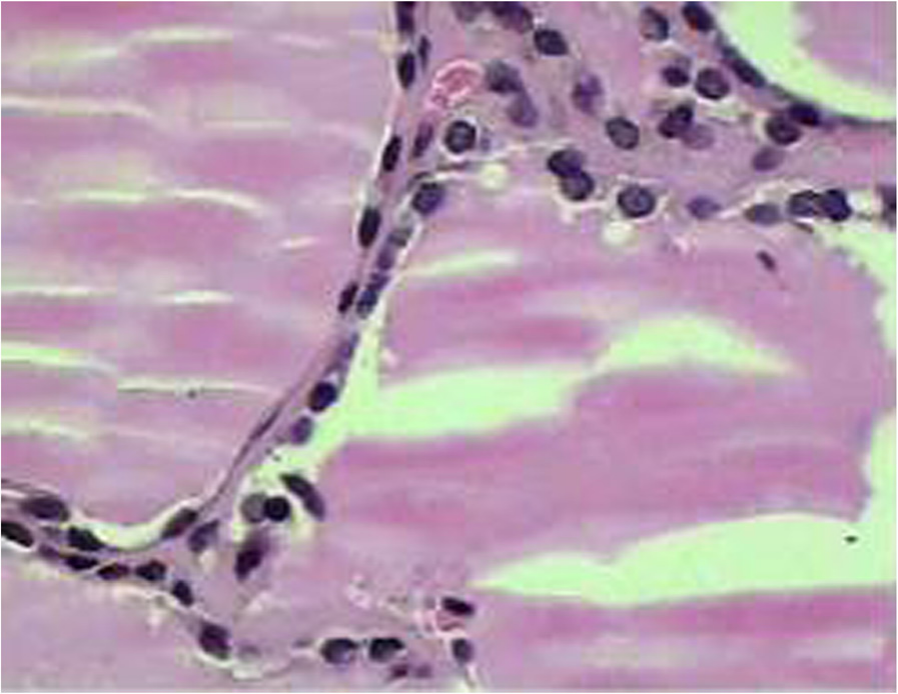
Microscopy of goiter showing no signs of goiter malignancy.

**Figure 3:**
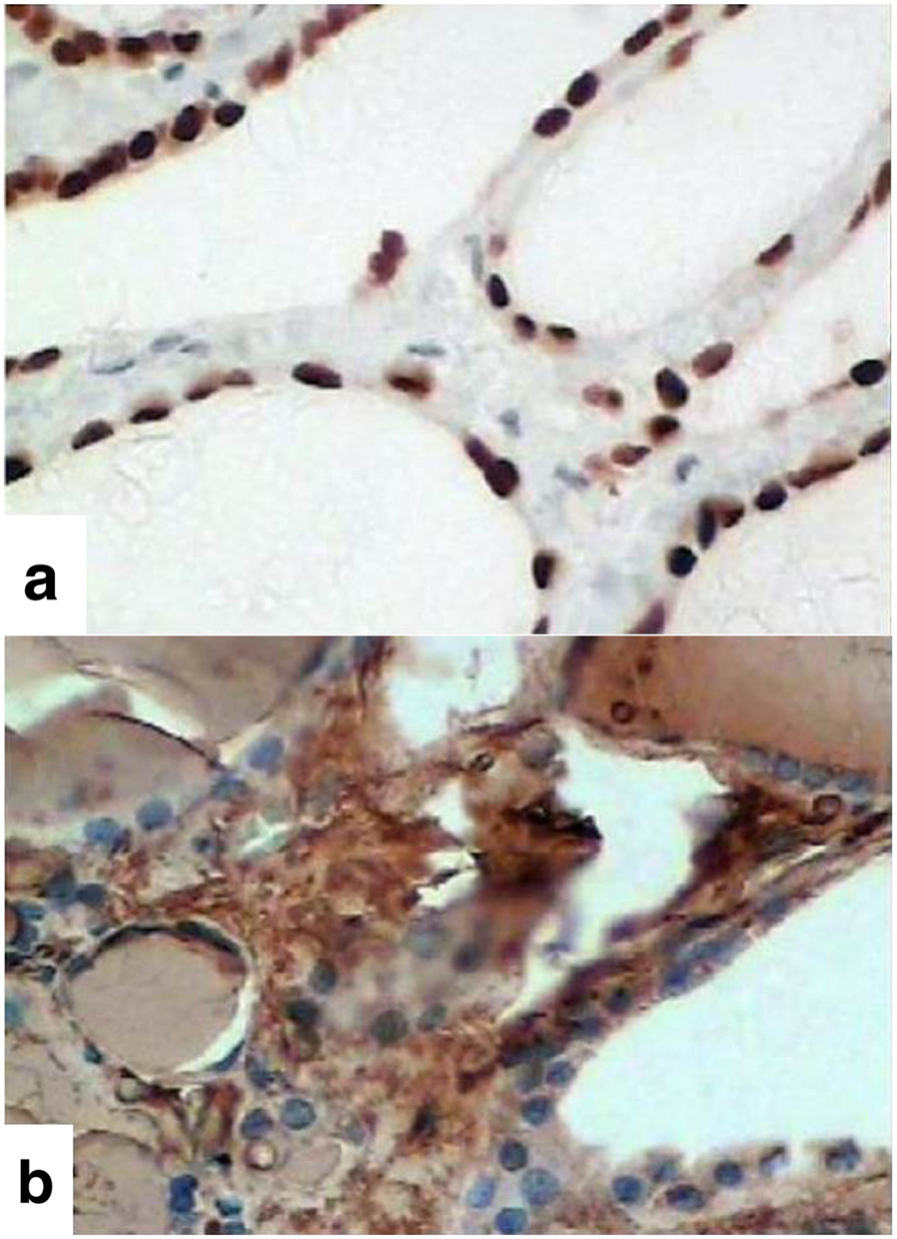
**Immunohistochemistry of nodules. a)** Expression of TTF-1; **b)** expression of thyroglobulin.

Microscopically, the goiter was composed of thyroid tissue with normo- and macroscopic follicles that contained colloid and a coated monolayer of cells with regular, uniform nuclei that were round to oval and had fine chromatin, as well as homogeneous eosinophilic cytoplasm. There were no papillary formations, psammoma bodies or nuclear atypia, such as clear core, slit or pseudo nuclear inclusions (Figure 2). The material did not have characteristics consistent with malignancy. Expression of thyroid transcription factor (TTF-1) and thyroglobulin on immunohistochemistry confirmed the thyroid origin of nodules (Figure 3). The findings corresponded to a parasitic thyroid nodule.

## Discussion

Thyroid tissue can be located in the lateral region of the neck under three circumstances. First, thyroid tissue can be present when there is mechanical deployment of the tissue after thyroid surgery or cervical trauma; second, when parasitic thyroid nodules are present without associated lymph nodes; and third, when there are metastases of thyroid tissue in lymph nodes [1].

The nodules are the result of a parasitic growth of extracapsular nodular thyroid waste that becomes separated from a preexisting nodular goiter. It has been proposed that mechanical action of the cervical muscles over a nodular goiter could cause the separation of these thyroid residues [4]. Portions of goiter that protrude through the fascia can be cut by muscle pressure on the thyroid. This explains why the thyroid gland and parasitic nodules have the same histology and parasitic thyroid nodules have no evidence of malignancy [5,6].

## Conclusion

We conclude that the parasitic thyroid nodule should be included in the differential diagnosis of lateral neck masses. The confirmation of a parasitic thyroid nodule requires that it is in the same fascial plane of the thyroid, has similar histology as the thyroid, and cannot be associated with lymph nodes. This benign condition is considered to be rare, and it can cause a serious dilemma if there is suspicion of an occult primary thyroid carcinoma. The diagnosis and differentiation of these nodules from metastatic adenopathies of differentiated thyroid carcinoma have important therapeutic and prognostic implications, and may prevent unnecessary medical examinations or treatments in the future.

## Consent

Written informed consent was obtained from the patient for publication of this case report and accompanying images. A copy of the written consent is available for review by the Editor-in-Chief of this journal.

## Competing interests

The authors declare that they have no competing interests.

## Authors’ contributions

JROF and TRN performed the surgery and clinical evaluation, and analyzed and reviewed all exams and the medical history of the patient regarding this pathology. Both authors read and approved the final manuscript.
